# Transcriptional and electrical identity in endothelial cells is orchestrated by intercellular coupling

**DOI:** 10.3389/fphys.2025.1662268

**Published:** 2025-11-04

**Authors:** Pía C. Burboa, Veronica Kuzdowicz, Stefany Ordenes, Helmuth A. Sánchez, Mauricio A. Lillo

**Affiliations:** ^1^ Department of Pharmacology, Physiology and Neuroscience, Rutgers-New Jersey Medical School, Newark, NJ, United States; ^2^ Centro Interdisciplinario de Neurociencia de Valparaíso, Facultad de Ciencias, Universidad de Valparaíso, Valparaíso, Chile; ^3^ Centro de Neurología Traslacional, Facultad de Medicina, Universidad de Valparaíso, Valparaíso, Chile; ^4^ Programa de Doctorado en Ciencias, Mención Neurociencia, Universidad de Valparaíso, Valparaíso, Chile

**Keywords:** endothelium, calcium, EDH, arteriole, resting membrane potential (RMP)

## Abstract

The membrane potential (Vm) of vascular cells is a fundamental determinant of vasomotor tone, particularly in resistance arteries and arterioles where precise electrical signaling ensures tissue perfusion and blood pressure regulation. While the electrophysiological role of vascular smooth muscle cells is well characterized, the bioelectrical contribution of endothelial cells (ECs)—especially their Vm dynamics, calcium responsiveness, and transcriptional plasticity—remains incompletely understood. Here, we combined intracellular microelectrode recordings, calcium imaging, RNAscope, and immunofluorescence to dissect the transcriptional and electrical landscape of ECs in murine mesenteric arterioles and primary cultures. ECs cultured in isolation exhibited a depolarized Vm (∼–25 mV), disrupted ion channel organization, and reduced expression of key regulators, including FOXO3, MEF2C, KCa2.3, KCa3.1, and Kir2.1. Functionally, these cells failed to hyperpolarize or elevate intracellular calcium in response to acetylcholine (ACh) or the KCa channel activator SKA-31. In contrast, electrically coupled ECs displayed a more negative and heterogeneous Vm (−65 mV to −40 mV), robust hyperpolarization, and markedly enhanced calcium responses. This phenotype correlated with spatially coordinated upregulation of ion channels and transcription factors, as shown by RNAscope and immunofluorescence, supporting the existence of a coupling-dependent transcriptional program that sustains endothelial bioelectrical competence. Strikingly, time-resolved heatmaps and 3D activity maps revealed that coupled EC generate synchronized calcium waves upon ACh stimulation, with greater ΔF/F_0_ amplitudes and spatiotemporal coordination compared to non-coupled counterparts. These organized calcium dynamics mirrored peripheral ion channel clustering and supported a hyperpolarization-competent phenotype. Consistently, a subpopulation (∼30%) of ECs in coupled conditions exhibited Vm values below −55 mV. Supporting our findings, endothelium removal in intact arterioles abolished the hyperpolarized Vm component and depolarized the vessel wall toward −30 mV, indicating that the endothelium plays a dominant role in setting arteriolar membrane potential. Altogether, our data uncover a transcriptionally regulated, calcium-sensitive, and electrically competent endothelial phenotype that critically depends on cell–cell coupling. We propose that connexin-mediated communication not only enables ionic and metabolic exchange but also synchronizes transcriptional programs that define endothelial identity and ensure vascular integration. Disruption of this electro-transcriptional coupling may represent an early hallmark of endothelial dysfunction and impaired vasodilatory conduction. These findings offer a mechanistic framework for identifying early-stage biomarkers and therapeutic targets aimed at preserving endothelial integrity in cardiovascular disease.

## Introduction

Arterioles are key regulators of peripheral vascular resistance and systemic blood pressure. Changes in vasomotor tone—dynamic alterations in vessel diameter—have a direct impact on blood pressure, contributing to hypertensive or hypotensive phenotypes with potentially severe consequences. Arterioles achieve this regulation through precise control of vascular tone, a process strongly influenced by the membrane potential (Vm) of their constituent cells, particularly vascular smooth muscle cells (VSMCs) and endothelial cells (ECs). While VSMCs are traditionally recognized for their role in regulating contractile tone, endothelial cells also contribute significantly—not only through the release of paracrine factors such as nitric oxide and prostacyclin but also via electrical signaling mechanisms that are essential for arteriole dilation and overall vasomotor regulation (Ledoux et al., 2008; Longden and Nelson, 2015; Nelson et al., 1990; Shimokawa and Godo, 2016).

Unlike electrically excitable cells such as neurons or cardiac myocytes, ECs are often considered “non-excitable” due to their lack of action potentials. Nevertheless, the resting membrane potential in ECs plays a pivotal role in modulating cellular behavior, particularly in regulating Ca^2+^ entry via store-operated or voltage-insensitive pathways, and in modulating the activity of ion channels such as Ca^2+^-activated potassium channels (K_Ca_). Changes in Vm can thus have profound effects on endothelial function, particularly in the context of endothelium-dependent hyperpolarization (EDH)-mediated vasodilation, which is prominent in small resistance vessels (Bagher and Segal, 2011; Garland et al., 2011; Jackson, 2022; Sandow et al., 2002).

Recent studies have revealed that the endothelium is not electrically homogeneous. Instead, subpopulations of endothelial cells can exhibit distinct electrophysiological phenotypes, likely reflecting both spatial organization and functional specialization (Siegl et al., 2005; Ulyanova and Shirokov, 2018). This heterogeneity becomes particularly important in understanding how electrical signals propagate along the endothelium and influence underlying VSMCs. Moreover, the extent of electrical coupling through gap junctions—primarily composed of connexin proteins like Cx37, Cx40, and Cx43 — is a critical determinant of whether local changes in Vm can spread and contribute to coordinated vasomotor responses (Behringer and Segal, 2012; Looft-Wilson et al., 2004).

A significant aspect of the endothelial contribution to vascular tone involves myoendothelial gap junctions (MEJs). These specialized structures establish direct electrical and chemical communication pathways between ECs and VSMCs, facilitating the coordination of vasomotor responses. Gap junctions at the MEJ provide a crucial route for the direct transfer of ions (e.g., Ca^2+^) and signaling molecules between the endothelium and smooth muscle, closely linked to the fine-tuned regulation of vasomotor function (Lemmey et al., 2020). The number of MEJs and associated gap junctions increases in distal arteries, correlating with a heightened reliance on EDH (Dora et al., 2003). However, whether changes in the resting membrane potential or in the heterogeneity of endothelial Vm affect EDH remains unknown.

In this study, we investigated membrane potential dynamics in both cultured mesenteric ECs and intact resistance vessels. We found that primary mesenteric ECs cultured in isolation exhibit a depolarized average resting membrane potential of approximately −25 mV. In contrast, when these ECs remain electrically coupled under the same culture conditions, they display greater heterogeneity in membrane potential (Vm), with subpopulations ranging from approximately −65 mV to −40 mV. This distribution closely mirrors the Vm observed in intact mesenteric arteries, where both ECs and vascular smooth muscle cells (VSMCs) show resting potentials within the same range. Notably, removal of the endothelium via air bubble disruption eliminates the hyperpolarized component (∼−65 mV) and shifts the overall Vm toward more depolarized values (∼−30 mV), providing strong evidence that the endothelium plays a key role in maintaining a hyperpolarized resting membrane potential in the vascular wall. Additionally, isolated ECs—despite being under identical environmental and culture conditions as their coupled counterparts—fail to generate hyperpolarizing responses upon stimulation with K_Ca_ channel activators. This functional deficit indicates that electrical coupling between ECs is not only critical for sustaining resting Vm but also essential for proper endothelial responsiveness. Collectively, these findings underscore a previously underappreciated role for cell-to-cell electrical communication in preserving endothelial function and regulating vascular tone. Disruption of this coupling may therefore contribute to endothelial dysfunction in pathological conditions, highlighting it as a potential target for therapeutic intervention.

## Results

### Endothelium controls electrical polarization of smooth muscle in mesenteric resistance arteries

**FIGURE 1:**
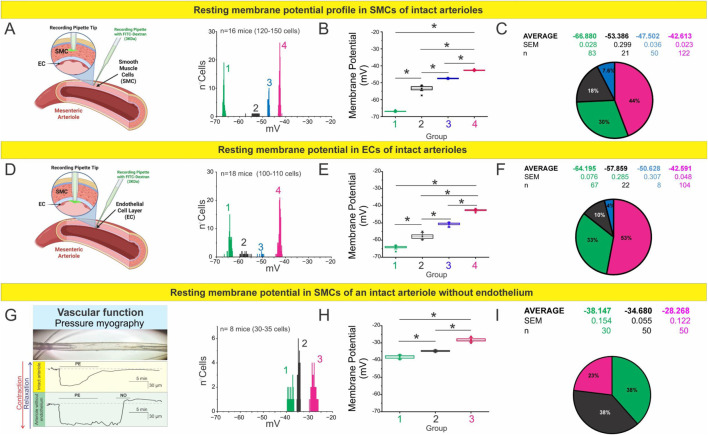
The endothelium regulates the membrane potential profile of mesenteric arterioles. **(A–C)** RMP profile of smooth muscle cells (SMCs) in intact mesenteric arterioles. **(A)** Schematic illustration of sharp electrode intracellular recordings in SMCs using Alexa Fluor 488–labeled pipettes. The histogram shows RMP distributions across 120–150 SMCs obtained from n = 16 mice, revealing four distinct membrane potential clusters. **(B)** Boxplot summarizing the average RMP of each cluster. Statistical analysis was performed using nested ANOVA followed by Tukey’s multiple comparison post test (p < 0.001). **(C)** Pie chart representing the relative proportion and mean ± SEM of each RMP cluster. **(D–F)** RMP profile of endothelial cells (ECs) in intact mesenteric arterioles. **(D)** Schematic of intracellular recordings targeting ECs. The histogram shows RMP distributions across 100–110 cells from n = 18 mice, identifying four distinct populations. **(E)** Boxplot summarizing average RMP values for each cluster. Nested ANOVA followed by Tukey post test (p < 0.001) confirmed significant differences among all groups. **(F)** Pie chart showing the relative distribution and mean ± SEM values of each subpopulation. **(G–I)** RMP profile of SMCs in arterioles after endothelial removal. **(G)** Representative pressure myography traces demonstrating preserved contractile and vasodilatory responses to phenylephrine (PE) and nitric oxide (NO), confirming the functional integrity of denuded vessels. **(H)** Histogram and boxplot of RMPs from 30 to 35 SMCs (n = 8 mice), analyzed by nested ANOVA followed by Tukey post test (p < 0.001). **(I)** Pie chart summarizing the relative proportions and mean ± SEM for each cluster, showing a depolarized profile compared to intact vessels, underscoring the critical role of the endothelium in establishing vascular electrical states.

Resting membrane potentials of both smooth muscle cells and endothelial cells were evaluated in intact mesenteric arteries, as shown in Figures 1A,C,E. For smooth muscle cells, measurements were obtained under pressure myography conditions at a luminal pressure of 60 mmHg. For endothelial cells, recordings were performed using *en face* preparations to allow direct access to the endothelial layer. In endothelial cells (Figures 1D,F), instead of observing a single, well-defined value, we detected a notable heterogeneity in the recorded resting potentials. These values could be categorized into four distinct subpopulations with mean membrane potentials of −64.2 mV (n = 66), −57.9 mV (n = 21), −50.6 mV (n = 8), and −42.6 mV (n = 107). As detailed in the Methods, these analyses were performed in 15–20 mice, with 3–5 arterioles analyzed per mesentery.

Interestingly, when we mechanically removed the endothelial layer (Figures 1G–I), the resting membrane potential of smooth muscle cells shifted toward more depolarized values. In this context, we identified three distinct subpopulations with average potentials of −38.1 mV (n = 30), −34.7 mV (n = 50), and −28.3 mV (n = 50). The functional integrity of the smooth muscle cells following endothelial denudation was confirmed by assessing their contractile response to phenylephrine (10 µM) and their relaxation response to a nitric oxide donor (SNAP 1 µM) (Figure 1G). These findings highlight the crucial role of the endothelium in maintaining the electrical stability of vascular smooth muscle cells under physiological conditions, establishing it as a fundamental determinant of resting membrane potential in intact vessels.

Despite the diversity of values, each subpopulation exhibited low internal variability, suggesting that the observed membrane potentials are not random but reflect the presence of discrete cellular states or subtypes. This, in turn, points to the likely contribution of molecular or structural substrates that define and stabilize these resting potential values within an apparently homogeneous tissue.

### Cell confluence influences resting membrane potential in endothelial cells via gap junction coupling

**FIGURE 2:**
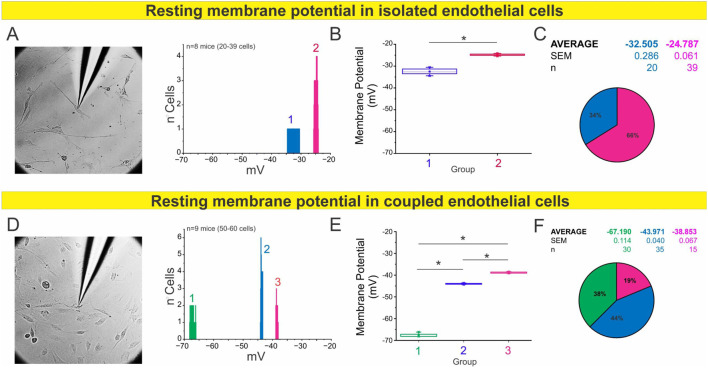
Isolated endothelial cells display a depolarized resting membrane potential profile. **(A)** Representative image showing sharp electrode impalement in isolated endothelial cells. The histogram depicts RMP distributions from 20 to 39 cells obtained from *n* = 8 mice, revealing two distinct membrane potential clusters. **(B)** Boxplot summarizing the average RMP of each cluster. Statistical analysis was performed using nested ANOVA followed by Tukey’s multiple comparison post test (*p* < 0.001). **(C)** Pie chart displaying the relative proportion and mean ± SEM of each RMP cluster. **(D–F)** RMP profile of coupled endothelial cells (cells forming gap junctions). **(D)** Representative image of intracellular recordings in coupled endothelial cells. The histogram shows RMP distributions across 50–60 cells from *n* = 9 mice, identifying three distinct subpopulations. **(E)** Boxplot summarizing the average RMP values for each group, analyzed by nested ANOVA followed by Tukey post test (*p* < 0.001). **(F)** Pie chart representing the relative distribution and mean ± SEM of each cluster, showing a more polarized profile compared with isolated cells, consistent with enhanced electrical coupling through gap junctions.

To further investigate whether intercellular coupling—presumably mediated by gap junction channels—affects the resting membrane potential of endothelial cells, we cultured endothelial cells isolated from mesenteric arteries under conditions of isolated and coupled cells (forming gap junctions) (Figures 2A–F, respectively). In coupled ECs more hyperpolarized membrane potentials were observed, with three distinguishable subgroups averaging −67.2 mV (n = 30), −44.0 mV (n = 35), and −38.9 mV (n = 15). In contrast, in isolated cells, where intercellular contacts are sparse, two relatively depolarized subpopulations were identified with average potentials of −32.5 mV (n = 20) and −24.8 mV (n = 39). Again, the low variability within each subgroup supports the notion of distinct electrophysiological states, likely determined by underlying molecular mechanisms.

**FIGURE 3:**
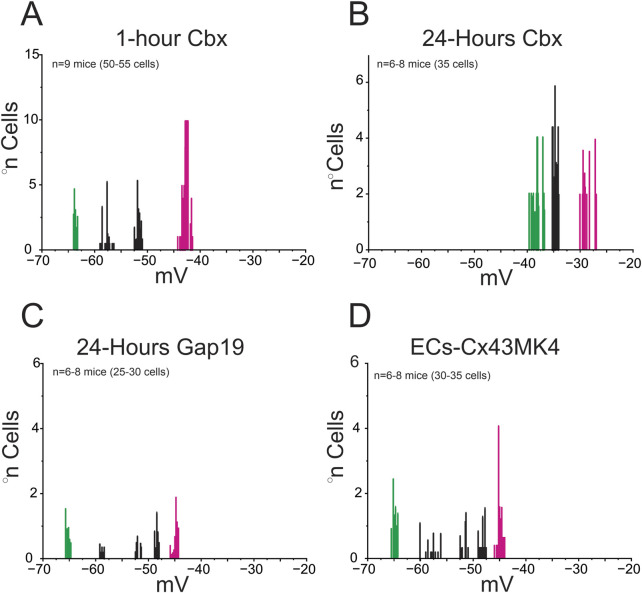
Gap junctions, but not hemichannels, regulate resting membrane potential profiles in coupled endothelial cells. Histograms show resting membrane potential (V_m_) distributions from primary mesenteric endothelial cells cultured under coupled conditions for 48 h. **(A)** Acute exposure to carbenoxolone (Cbx, 1 h) did not alter Vm relative to untreated controls (see Figure 2), indicating that short-term gap-junction blockade is insufficient to modify baseline electrical properties. **(B)** In contrast, prolonged Cbx treatment (24 h) markedly shifted the distribution toward depolarized values, reflecting impaired electrical coupling. **(C)** Selective inhibition of Cx43 hemichannels with Gap19 (24 h) did not significantly affect Vm, showing that hemichannel activity is dispensable for basal regulation. **(D)** Endothelial cells from Cx43MK4 mice, in which gap-junction communication is preserved but hemichannel activity is impaired, displayed Vm profiles similar to controls. Each V_m_ subpopulation was statistically distinct, as determined by nested ANOVA followed by Tukey’s multiple-comparison post test (p < 0.001). Data were obtained from *n* = 6–9 mice (25–55 cells; 2–5 covers per *n*). Collectively, these results indicate that gap-junctional coupling, rather than hemichannel activity, is required to maintain endothelial membrane potential under coupled conditions.

Because our initial analyses revealed that endothelial confluency determines the distribution of resting potential, we next tested whether this effect depends on connexin-based communication. To assess the contribution of connexin channels, we treated coupled ECs with carbenoxolone (Cbx, 50 μM, a general connexin blocker). Acute blockade (1 h) did not significantly alter the distribution of resting potentials, which remained comparable to untreated coupled cells (Figure 3A). However, prolonged Cbx exposure (24 h) markedly reduced the hyperpolarized subpopulations and shifted most cells toward less negative values (∼−40 to −30 mV; Figure 3B). These findings indicate that sustained inhibition of connexin-mediated communication collapses the electrical heterogeneity normally observed in coupled.

Because Cbx blocks both gap junctions and hemichannels, we next used two complementary approaches to specifically address the role of Cx43 hemichannels. Selective inhibition with Gap19 (50 μM, 24 h) reproduced the loss of the most negative states, yielding two main peaks at ∼ −60 and −40 mV (Figure 3C). Similarly, endothelial cells from Cx43MK4 knock-in mice (in which four serine residues phosphorylated by MAPK (S255, S262, S279, and S282) are replaced by alanines), in which gap junctional coupling is preserved but hemichannel activity is selectively impaired (Freitas-Andrade et al., 2019), also displayed redistribution toward depolarized values with peaks at ∼ −60, −50, and −40 mV (Figure 3D).

These findings indicate that intercellular coupling through gap junctions is a key determinant of endothelial resting membrane potential. Under high-confluence conditions, electrotonic interactions stabilize hyperpolarized states, whereas low confluency favors depolarized profiles, underscoring that endothelial electrical behavior is defined not only by intrinsic properties but also by the degree of cellular connectivity.

### Cell density modulates the amplitude and coordination of endothelial calcium responses

Given that cell-to-cell coupling may regulate not only the resting membrane potential but also intracellular calcium dynamics, we next evaluated calcium signaling responses in endothelial cells under varying confluence conditions. Endothelial cells isolated from mesenteric arteries were cultured at low or high confluence, loaded with the calcium-sensitive dye Fluo-4 AM, and stimulated with acetylcholine (1 µM). Intracellular calcium levels were recorded using time-lapse fluorescence imaging.

**FIGURE 4:**
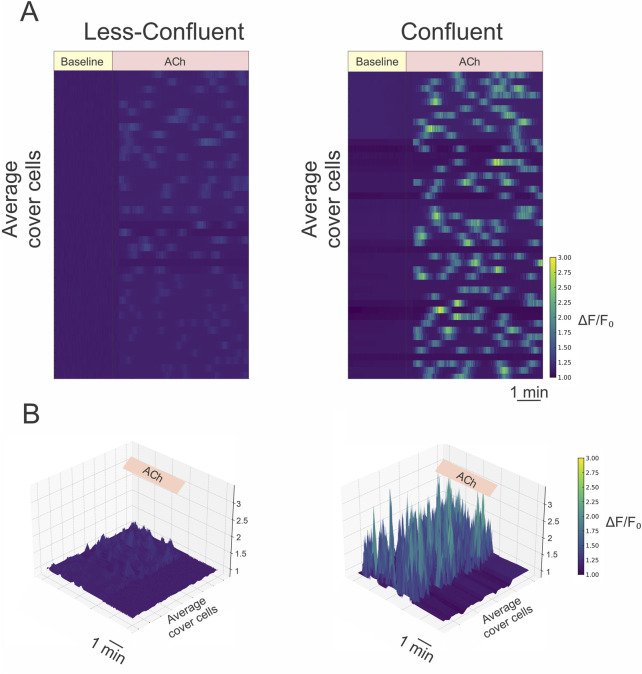
Coupled cells exhibit enhanced and coordinated calcium responses to acetylcholine stimulation. **(A)** Representative heatmaps illustrating temporal changes in intracellular calcium levels (ΔF/F_0_) in non-coupled (left) and coupled (right) endothelial cells loaded with 5 μM Fluo-4 AM. Cells were stimulated with 1 μM acetylcholine (Ach), as indicated. Each row represents the calcium signal from an individual cell, and images were acquired every 2 s. The heatmap reveals a pronounced increase in both amplitude and synchronicity of calcium responses in coupled compared to non-coupled conditions. **(B)** Corresponding three-dimensional surface plots of the same recordings shown in **(A)**, highlighting the spatiotemporal dynamics of calcium activity. Coupled endothelial cells demonstrate robust, coordinated calcium waves in response to Ach, in contrast to the blunted and heterogeneous responses observed in non-coupled cells. Statistical analysis confirmed that the overall calcium responses were significantly different between non-coupled and coupled cells, as determined by nested ANOVA followed by Tukey’s post test (p < 0.001).

As shown in Figures 4A,B, coupled ECs—which promote physical proximity and likely increased intercellular coupling via gap junctions—exhibited significantly greater calcium responses to acetylcholine compared to their low-confluence counterparts. The increase in intracellular calcium was not only more pronounced in amplitude but also temporally dynamic, with repetitive transient elevations persisting throughout the 5-minute exposure period. These findings suggest that endothelial cells in dense coupled retain their sensitivity to cholinergic stimulation over time and do not exhibit rapid desensitization under these conditions.

In contrast, low-confluence ECs showed weaker and less coordinated responses, consistent with limited intercellular communication. Importantly, we also observed a higher degree of synchrony in calcium transients among adjacent cells in coupled ECs, indicative of coordinated calcium signaling across the monolayer. This spatial coordination further supports the presence of functional gap junctional communication enabling calcium signal propagation between endothelial cells.

Together, these results demonstrate that endothelial cell density and intercellular connectivity critically influence the amplitude, frequency, and spatial coordination of calcium responses to acetylcholine. These findings reinforce the concept that gap junction-mediated coupling is not only essential for electrical synchronization but also for dynamic calcium signaling within the endothelial layer—highlighting a key role for intercellular communication in shaping the endothelial response to physiological stimuli.

### Functional endothelial coupling is essential for KCa-Driven EDH in resistance vasculature

The pronounced hyperpolarization detected in a subset of endothelial cells *in situ* (Figure 1C) and in highly coupled ECs led us to investigate whether calcium-activated potassium channels (KCa2.3 and KCa3.1) contribute to this response and whether their activation depends on intercellular coupling. These channels, which open in response to rises in intracellular calcium, are essential effectors of endothelium-dependent hyperpolarization (EDH) — a key mechanism of vasodilation in resistance arteries, particularly under conditions where nitric oxide (NO) bioavailability is impaired (Shimokawa and Godo, 2016). To test this, we assessed membrane potential changes in endothelial cells cultured under isolated or coupled cells, using sharp microelectrode impalements to record resting membrane potential (V_m_) before and after stimulation with either the selective KCa2.3/KCa3.1 activator SKA-31 (1 µM) (Köhler et al., 2016) or acetylcholine (1 µM), a physiological stimulus that mobilizes endothelial Ca^2+^(Félétou, 2016).

**FIGURE 5:**
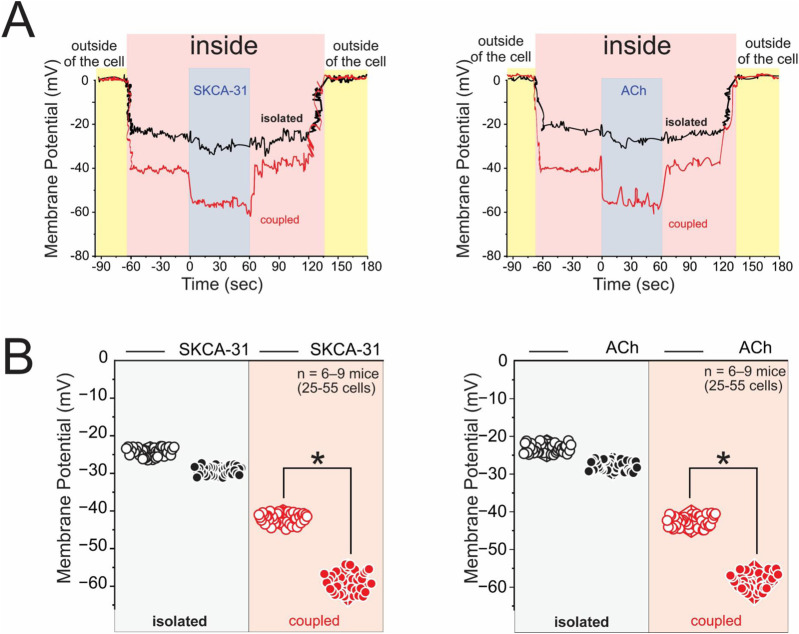
Endothelial cell coupling amplifies membrane hyperpolarization in response to KCa channel activation and acetylcholine stimulation. **(A)** Representative sharp microelectrode recordings of membrane potential (V_m_) from isolated (black) and coupled (red) endothelial cells. The yellow-shaded regions represent the period before the pipette entered the cell (“outside”), while the pink-shaded region indicates the recording after impalement (“inside”). Cells were stimulated with either 1 μM SKA-31 (left), an activator of small- (KCa2.3) and intermediate-conductance (KCa3.1) Ca^2+^-activated K^+^ channels, or 1 μM acetylcholine (ACh, right). Coupled endothelial cells exhibited a pronounced hyperpolarization following stimulation, whereas isolated cells showed minimal changes. **(B)** Quantification of resting membrane potential at baseline and after stimulation with either SKA-31 or ACh. Data are shown for isolated (black circles) and coupled (red circles) endothelial cells. Statistical comparisons were performed using nested ANOVA followed by Tukey’s multiple-comparison post test, confirming that coupled ECs display significantly greater hyperpolarization in response to both SKA-31 and ACh (p < 0.001). Data were obtained from *n* = 6–9 mice (25–55 cells; 2–5 covers per *n*).

As shown in Figure 5A, SKA-31 induced a robust and reversible hyperpolarization in coupled endothelial cells, with a rapid membrane shift of approximately 25 mV that returned to baseline upon washout. In contrast, isolated cells displayed only a minimal deflection in V_m_ under the same conditions. Similarly, acetylcholine triggered a hyperpolarization of comparable magnitude and kinetics in coupled cells, while isolated cells again showed little to no response. Quantitative analysis from a large cohort of cells (Figure 5B) confirmed that the amplitude of the hyperpolarizing response was significantly greater in coupled cells compared to isolated cells for both SKA-31 and ACh.

### Cx43 and KCa channel localization varies with endothelial density

**FIGURE 6:**
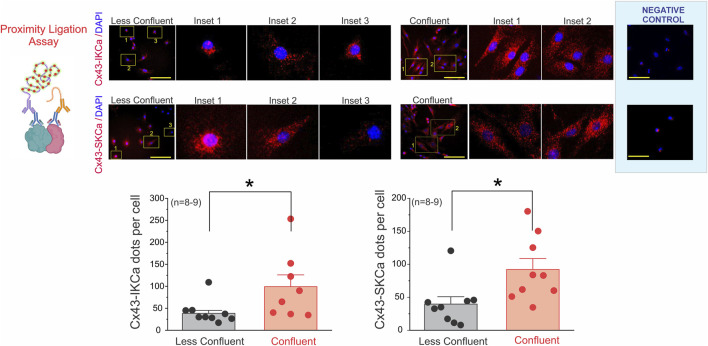
Endothelial confluency modulates the spatial distribution of Cx43–KCa channel interactions. Representative proximity ligation assay (PLA) images showing Cx43–IKCa (top panels) and Cx43–SKCa (middle panels) interactions in endothelial cells cultured under low-vs. high-confluency conditions. Under sparse conditions, PLA puncta were few and mainly localized to perinuclear regions, whereas coupled displayed a marked redistribution toward intercellular junctions (yellow boxes, magnified insets). Nuclei were counterstained with DAPI (blue). Quantification of PLA signals (bottom graphs) demonstrated significantly higher numbers of Cx43–IKCa and Cx43–SKCa puncta per cell in coupled cultures compared with less coupled cells. Data are presented as mean ± SEM; *n* = 8–9 independent experiments, with 2–5 coverslips per condition and 50–100 cells analyzed per experiment. Statistical significance was assessed using nested one-way ANOVA, with cells nested within coverslips and coverslips nested within experiments (*P* < 0.001). Scale bars: 50 µm (overview panels) and 20 µm (insets).

We previously demonstrated that Cx43 hemichannels are required for TRPV4- and acetylcholine-induced Ca^2+^ entry in endothelial cells, thereby driving hyperpolarization and EDH in resistance arteries (Burboa et al., 2025). Building on this, we reasoned that the spatial arrangement of Cx43 relative to IK_Ca_ and SK_Ca_ channels could determine the efficiency with which hemichannel-mediated Ca^2+^ influx is translated into K_Ca_ activation. Proximity ligation assays (PLA) revealed that under low-confluency conditions (Figure 6), Cx43–IKCa and Cx43–SKCa interactions were sparse and predominantly perinuclear, averaging 38.5 ± 6.7 (n = 9, 2–5 covers per n) and 39.7 ± 11.2 (n = 9, 2–5 covers per n) dots/cell, respectively. By contrast, coupled cells exhibited a marked redistribution of PLA puncta toward intercellular junctions, with significantly higher counts of 99.3 ± 26.7 (n = 8, 2–5 covers per n) for IKCa and 92.3 ± 16.4 (n = 9, 2–5 covers per n) for SKCa (P < 0.05 vs. less coupled). These findings demonstrate that endothelial density governs the nanoscale organization of Cx43–KCa channel complexes, providing a structural framework through which Ca^2+^ entry via hemichannels can be efficiently coupled to K_Ca_ activation and sustained EDH.

### Intercellularly coupled endothelial cells display distinct transcriptional profiles compared to isolated cells

To gain mechanistic insight into the molecular programs underlying the electrical heterogeneity and coupling-dependent hyperpolarization observed in endothelial cells, we investigated the spatial expression of transcription factors known to regulate ion channel activity, calcium signaling, and endothelial phenotype. Among the candidate regulators, FOXO3 and MEF2C were selected based on their established roles in vascular biology and transcriptional control of ion channel expression. FOXO3 is a redox-sensitive factor involved in maintaining endothelial quiescence and has been implicated in the regulation of potassium channels relevant to electrical responses (Zhao and Liu, 2021). MEF2C, activated by sustained intracellular calcium signals, has been linked to the transcriptional control of calcium-activated potassium channels (KCa), particularly in the context of angiogenesis and vascular remodeling.

Given the well-established role of KCa2.3 and KCa3.1 channels in mediating endothelium-dependent hyperpolarization (EDH) in resistance arteries—and our prior observations that endothelial confluency influences both calcium signaling and the spatial organization of Cx43–KCa channel complexes—we hypothesized that differential expression of FOXO3 and MEF2C might contribute to the transcriptional programming underlying endothelial electrical heterogeneity.

**FIGURE 7:**
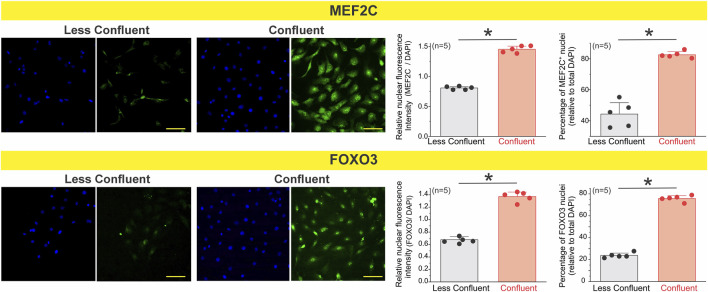
Nuclear accumulation of the transcription factors MEF2C and FOXO3 increases in coupled endothelial cells. Primary mesenteric endothelial cells were cultured at low or high confluence and immunostained for MEF2C and FOXO3. Representative images show reduced nuclear localization of both transcription factors under less coupled conditions and marked nuclear enrichment at confluence. Quantification was performed on ∼200–300 cells per condition, across 3 coverslips per culture, for a total of n = 5 independent cultures. Each biological replicate (n) represents the averaged value of its three coverslips. Coupled endothelial cells exhibited a significantly greater percentage of MEF2C- and FOXO3-positive nuclei and higher normalized nuclear fluorescence intensity compared with less coupled cultures. These results indicate that transcription factor nuclear accumulation is enhanced when mesenteric endothelial cells establish intercellular coupling. Statistical significance was assessed using nested one-way ANOVA, with cells nested within coverslips and coverslips nested within experiments (*P* < 0.001).

To test this, we examined the expression of FOXO3 and MEF2C in primary endothelial cells cultured under low- and high-density conditions (Figure 7). Immunofluorescence analysis revealed that both transcription factors were markedly upregulated in coupled cells, where cells exhibited an elongated, aligned morphology consistent with a quiescent and coupled phenotype. In contrast, less coupled cells showed reduced expression of FOXO3 and MEF2C, alongside a more spread and isolated morphology. These findings suggest that endothelial confluency enhances activation of calcium- and redox-sensitive transcriptional programs, which may in turn promote the expression or functional organization of ion channels critical for coordinated calcium signaling and hyperpolarization in response to physiological stimuli such as acetylcholine.

**FIGURE 8:**
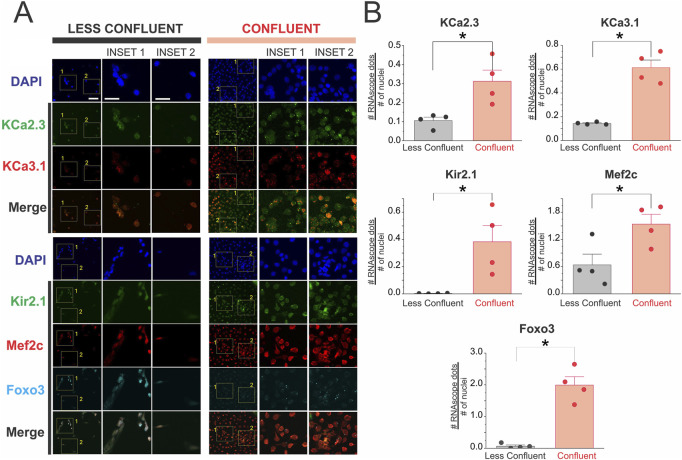
**(A,B)** Endothelial coupling enhances the transcriptional expression of potassium channels and key transcription factors. RNAscope *in situ* hybridization was performed in primary endothelial cell cultures under low-density (Less Coupled) and high-density (Coupled) conditions to assess mRNA expression of ion channels and transcriptional regulators involved in endothelial electrical identity. Representative maximum projection images show increased transcript abundance for the inward rectifier potassium channel Kir2.1, the small- and intermediate-conductance calcium-activated potassium channels KCa2.3 and KCa3.1, and the transcription factors Mef2c and Foxo3 in coupled compared to sparsely seeded cultures. Insets 1 and 2 highlight selected regions of interest with increased punctate signal intensity in the coupled condition, indicating active transcription and spatial clustering of target mRNAs. Scale bars = 100 μm (left insets), 50 μm (right insets). Statistical significance was assessed using nested one-way ANOVA, with cells nested within coverslips and coverslips nested within experiments (*P* < 0.001). Images are representative of n = 4 independent experiments.

To investigate whether endothelial confluency shapes the transcriptional programs underlying electrical identity and hyperpolarization capacity, we performed RNAscope® *in situ* hybridization targeting transcripts for key ion channels and transcriptional regulators (Figure 8A). Under sparse, less coupled conditions, endothelial cells displayed low and scattered puncta for KCa2.3, KCa3.1, and Kir2.1, consistent with a poorly organized transcriptional profile for hyperpolarizing machinery. Likewise, signals for the transcription factors Mef2c and Foxo3 were minimal and distributed heterogeneously across nuclei.

In contrast, coupled cells exhibited a marked upregulation of punctate mRNA signal across all examined targets. Robust expression of Kir2.1 was detected broadly throughout coupled cells, suggesting increased transcription of inward-rectifier K^+^ channels that stabilize the endothelial resting membrane potential and facilitate electrical coupling (Figures 8A,B).

KCa2.3 and KCa3.1 transcripts were also significantly enriched under coupled endothelial cells conditions, consistent with transcriptional priming of Ca^2+^-activated K^+^ channels that mediate hyperpolarizing responses. The parallel induction of these two conductances implies coordinated transcriptional remodeling toward an enhanced capacity for endothelium-dependent hyperpolarization (Figures 8A,B).

Importantly, transcription factors with recognized roles in endothelial differentiation and ion channel regulation, including Mef2c and Foxo3, were also strongly elevated in coupled cells. Foxo3 puncta were frequently clustered within nuclear and perinuclear regions, whereas Mef2c expression was more broadly distributed, supporting a transcriptionally active state (Figures 8A,B). These results establish a direct association between endothelial confluency and the transcriptional enrichment of both hyperpolarizing ion channels and their upstream transcriptional regulators.

To determine whether intercellular gap-junctional communication contributes to the transcriptional program underlying endothelial electrical identity, we performed RNAscope® in coupled cells exposed to two structurally distinct connexin channel blockers: oleamide (50 μM, 6 h) and carbenoxolone (50 μM, 6 h). Vehicle-treated ECs coupled conditions exhibited robust punctate signals for KCa2.1, KCa2.3, KCa3.1, Foxo3, and the constitutive endothelial markers Cdh5 and Gja1. In contrast, pharmacological inhibition of gap junction channels markedly reduced transcript abundance across all tested targets.

**FIGURE 9:**
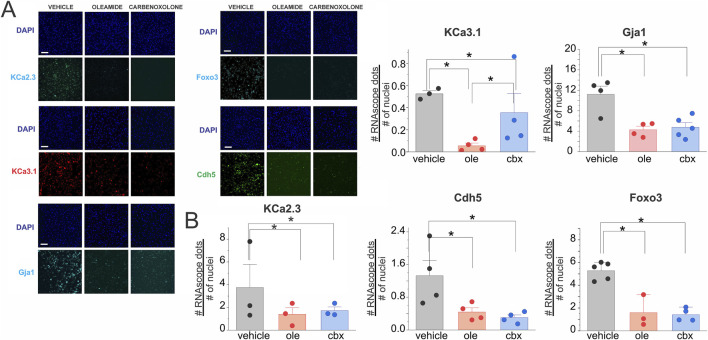
Pharmacological inhibition of intercellular gap junction channels decreases transcriptional expression of potassium channels, Foxo3, and constitutive endothelial markers. **(A)** RNAscope® *in situ* hybridization was performed in primary endothelial cell cultures under coupled conditions, treated with vehicle (0.1% v/v ethanol), oleamide (50 μM, 6 h), or carbenoxolone (50 μM, 6 h). **(A)** Representative images show mRNA transcripts for the calcium-activated potassium channels KCa2.3 and KCa3.1, and the gap junction protein Gja1, transcription factor Foxo3 and the constitutive endothelial marker Cdh5. Nuclei were counterstained with DAPI. **(B)** Quantification of RNAscope® signals demonstrates a significant reduction in transcript abundance of KCa2.3, KCa3.1, Gja1, Cdh5, and Foxo3 in cells treated with gap-junction blockers compared with vehicle. Values are expressed as the number of RNAscope® dots per nucleus, averaged across three randomly selected fields per condition. Data are presented as mean ± SD from *n*= 4-3 independent experiments, with 2–5 cover slips. Statistical significance was assessed using nested one-way ANOVA, with cells nested within coverslips and coverslips nested within experiments (*P* < 0.001).

Both oleamide and carbenoxolone decreased the expression of small- and intermediate-conductance Ca^2+^-activated K^+^ channels, which are central to endothelial hyperpolarization. Notably, inhibition of gap-junctional coupling also suppressed Foxo3, a transcription factor previously shown to be enriched under coupled conditions, as well as Cdh5 (VE-cadherin) and Gja1 (Cx43), two canonical endothelial identity markers (Figure 9).

These results indicate that the transcriptional reinforcement of electrical and endothelial identity observed under coupled conditions requires functional intercellular coupling. By disrupting gap junction communication, endothelial cells downregulate both ion channel and transcription factor expression, suggesting that electrical continuity through connexin-based channels provides a feedback signal necessary to sustain transcriptional programs for hyperpolarization competency and vascular quiescence.

## Discussion

**FIGURE 10:**
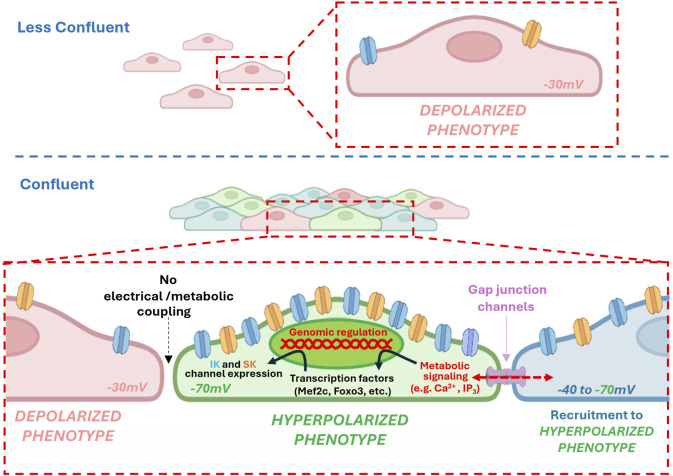
Intercellular gap junction channels drive transcriptional expression of potassium channels and differentiation markers, thereby establishing the functional and electrical identity of endothelial cells. Under conditions of reduced intercellular coupling—such as low confluence or in the presence of gap junction blockers—metabolic signaling required for endothelial differentiation is diminished. This is associated with reduced expression of hyperpolarizing potassium channels and a depolarized resting membrane potential. By contrast, high confluence promotes intercellular coupling and facilitates the diffusion of metabolic signals that regulate transcriptional programs, leading to the upregulation of hyperpolarizing potassium channels and other constitutive genes (e.g., VE-Cadherin, Connexin-43, among others). This recruitment of neighboring cells from a depolarized to a hyperpolarized state helps establish collective electrical identity. Importantly, local loss of coupling within coupled may account for the heterogeneity in electrical phenotypes observed among endothelial cells.

Our findings uncover a transcriptionally regulated bioelectrical program in the endothelium that is critically governed by cell confluency and intercellular coupling. Coupled endothelial coupled exhibited a hyperpolarization-competent phenotype characterized by more negative and heterogeneous resting membrane potentials, enhanced calcium signaling, and peripheral clustering of KCa channel complexes. This functional state was associated with upregulation of key transcription factors (FOXO3, MEF2C) and ion channels (KCa2.3/Kcnn3, KCa3.1/Kcnn4, and Kir2.1/Kcnj2), as revealed by RNAscope *in situ* hybridization. In contrast, sparsely seeded or uncoupled endothelial cells showed a marked loss of electro-transcriptional identity, displaying depolarized membrane potentials, diminished calcium responses, and disorganized ion channel localization. *In vivo* intracellular recordings further revealed discrete subpopulations of hyperpolarized endothelial cells in intact arterioles—a heterogeneity that was lost upon endothelial denudation. These data support a model in which endothelial coupling synchronizes both electrical and transcriptional states across the monolayer, likely via calcium- and IP_3_-dependent signaling. Importantly, we also demonstrate that endothelial electrical identity exerts dominant control over vascular tone, as endothelial removal significantly depolarized underlying smooth muscle cells. Together, these results establish endothelial confluency and coupling as central regulators of vascular bioelectric homeostasis (Figure 10).

Beyond their classical role as passive conduits for ions and small molecules, we propose that connexins function as active regulators of endothelial identity by synchronizing not only electrical signals but also transcriptional programs. The observed peripheral co-localization of Cx43 with KCa channels under coupled conditions suggests the formation of structured signaling microdomains that enable efficient calcium influx and membrane hyperpolarization. Importantly, this coupling likely facilitates the coordinated spread of intracellular messengers such as Ca^2+^ and IP_3_, which are known to influence gene expression via activation of transcription factors like MEF2C and FOXO3 (Dolmetsch et al., 1998; Hogan et al., 2003). Thus, gap junction-mediated communication may serve a dual role: enabling rapid electrical signaling and enforcing transcriptomic synchronization across the endothelial monolayer. This would ensure that cells maintain a unified, functional phenotype akin to that observed in intact vessels, optimizing the capacity for conducted vasodilation and vascular homeostasis. Loss of such coupling—whether through inflammation, oxidative stress, or physical disruption—not only impairs signal propagation but may trigger a shift toward a transcriptionally fragmented endothelium, increasing the risk for endothelial dysfunction, impaired regeneration, and pathological remodeling (Higashi, 2022; Shaito et al., 2022).

Our findings suggest that endothelial cell–cell contact serves as a critical upstream regulatory signal that activates a transcriptional program essential for sustaining bioelectrical competence. RNAscope and immunofluorescence analyses revealed that key regulators of endothelial homeostasis—including the transcription factors FOXO3 and MEF2C, and the calcium-activated potassium channels KCa2.3 and KCa3.1—are markedly upregulated in coupled. Functionally, these cells display robust membrane hyperpolarization in response to both acetylcholine and SKCa/IKCa channel activation (SKA-31), along with higher amplitude and spatially coordinated calcium responses. These features are consistent with the acquisition of a differentiated, electrically responsive endothelial phenotype. In contrast, isolated or sparsely seeded endothelial cells exhibit a depolarized, electrically silent state characterized by reduced expression of Kir2.1, disorganized ion channel profiles, and significantly diminished calcium influx—features indicative of disrupted electro-transcriptional coupling and a shift toward a less mature endothelial identity.

These observations are supported by prior work demonstrating that FOXO3 and MEF2C are sensitive to intracellular calcium and redox signals and govern transcriptional programs involved in endothelial quiescence, oxidative resistance, and ion channel expression (Lin and Beal, 2006; Potente et al., 2005). Similarly, the pivotal role of KCa channels in endothelium-derived hyperpolarization (EDH) and vasodilator signaling has been well established (Garland and Dora, 2017; Köhler et al., 2016). Importantly, our electrophysiological and transcriptional data reveal that confluency-dependent upregulation of Kir2.1 stabilizes the resting membrane potential in coupled cells, enabling them to respond more effectively to hyperpolarizing stimuli—a property lost in non-coupled conditions. These findings reinforce the notion that electrical phenotype in endothelial cells is not static, but rather dynamically shaped by the degree of intercellular connectivity and transcriptional activation of ion channels.

Together, these data highlight the necessity of considering endothelial confluency, coupling state, and transcriptional context as fundamental parameters in electrophysiological studies. Experimental models relying on isolated or sparsely seeded endothelial cells may underestimate the physiological capacity for electrical integration, signal amplification, and coordinated calcium responses. As our data show, the more coupled and coupled the endothelial monolayer, the more faithfully it recapitulates the bioelectrical behavior of intact vessels, including negative resting Vm, synchronous calcium dynamics, and responsiveness to vasoactive agonists (Bagher and Segal, 2011; Behringer and Segal, 2017; Straub et al., 2014). These findings support a model in which structural organization and contact-dependent transcriptional programs converge to establish a hyperpolarization-competent endothelium optimized for vascular signal propagation and homeostasis.

Electrophysiological measurements in intact arterioles revealed the existence of discrete subpopulations of hyperpolarized endothelial cells. These cells may serve as initiators or amplifiers of electrical signals, enabling the regenerative, non-decremental propagation of vasodilatory responses along the vessel axis via gap junctional coupling. This observation aligns with seminal work by (Figueroa and Duling, 2009), who demonstrated that focal electrical stimulation of cremaster arterioles triggers endothelium-dependent, TTX-sensitive conducted dilation mediated by voltage-gated sodium (Naᵥ1.2, Naᵥ1.6) and calcium (Caᵥ3.2) channels. Their findings provided physiological evidence that endothelial cells can behave as electrically excitable elements, capable of sustaining long-range vasomotor communication.

Consistent with this concept, *in vivo* intracellular recordings from murine cremaster arterioles (Siegl et al., 2005) and skeletal muscle arterioles (Ulyanova and Shirokov, 2018) have confirmed that arterioles exhibit a broad, bimodal distribution of resting membrane potentials, with a subset reaching values as negative as −80 mV. This level of polarization is classically associated with vascular smooth muscle cells yet appears to be achieved by a functionally specialized population of endothelial cells. Such cells may form the electrical backbone of the endothelium, coordinating fast, long-distance signaling via gap junctions, and potentially functioning as “endothelial pacemakers.”

Together, these studies support a unifying model in which endothelial electrical heterogeneity is not merely a byproduct of cell isolation or stochastic noise, but a regulated feature of vascular architecture. Our data expand on this by linking the emergence of this hyperpolarized phenotype to endothelial confluency and transcriptional programming—including upregulation of Kir2.1, SK channels, and redox-sensitive transcription factors like FOXO3 and MEF2C—thus defining a previously underappreciated axis of electrical specialization in resistance vessels.

Importantly, our study builds upon this concept by identifying a mechanistic link between transcriptional identity and electrical phenotype. Specifically, we show that hyperpolarized endothelial subpopulations are transcriptionally enriched for FOXO3, MEF2C, and calcium-activated potassium channels (KCa2.3, KCa3.1)—factors known to promote a bioelectrically competent state. Quantitative analysis across >200 individual endothelial cells revealed that approximately 30% of the population exhibited significantly more negative resting membrane potentials, a proportion sufficient to generate and propagate depolarizing signals capable of activating voltage-dependent Na^+^ and Ca^2+^ channels.

This degree of electrical heterogeneity may represent a physiological basis for regenerative conduction, whereby a specialized subset of transcriptionally primed, hyperpolarized endothelial cells orchestrate long-distance signaling in resistance arteries. Notably, focal activation of voltage-gated Na^+^ channels (e.g., Naᵥ1.2/1.6), as previously shown by Figueroa and Duling, can initiate this process; and as further demonstrated by (Lillo et al., 2018), such depolarizing events may secondarily activate the reverse mode of the Na^+^–Ca^2+^ exchanger (NCXrm), driving Ca^2+^ influx and downstream production of nitric oxide and EDH in intact resistance arteries. This mechanism underscores how subtle local shifts in membrane potential—initiated by discrete endothelial subsets—can trigger powerful amplification loops involving both electrical and calcium-dependent pathways, effectively integrating transcriptional, ionic, and signaling layers to sustain vasodilatory responses.

Such subpopulations may act as electrical “pacemakers” of the microcirculation, supporting both upstream and downstream vasodilation, especially under conditions of increased metabolic demand. Disruption of this heterogeneity—through endothelial denudation, inflammation, or oxidative stress—could lead to a loss of regenerative potential, attenuated signal propagation, and impaired flow distribution, as has been described in models of hypertension and diabetes (Behringer and Segal, 2012; Shi and Vanhoutte, 2009). These findings emphasize the functional relevance of endothelial electrical diversity and support the notion that restoring or preserving this heterogeneity may have therapeutic implications for vascular disorders characterized by microvascular dysfunction.

Beyond the vasculature, endothelial bioelectrical properties may influence cardiac excitability and rhythmogenesis. Endocardial endothelial cells modulate action potential propagation in cardiomyocytes via electrotonic or paracrine communication (Brutsaert, 2003; Narmoneva et al., 2004). Loss of FOXO3, KCa channel expression, or Cx43 coupling could disrupt this balance, potentially facilitating arrhythmogenic substrates, especially under ischemic or inflammatory stress. These findings broaden the scope of endothelial electrical dysfunction from vasomotor tone to cardiac arrhythmogenesis and myocardial perfusion mismatch.

The implications also extend to cancer biology and atherosclerosis. Tumor vasculature often comprises poorly organized, leaky, and electrically uncoupled endothelial cells, which may lack the coordinated calcium and membrane potential signaling required for controlled perfusion and barrier function (Goel et al., 2011). Similarly, early stages of atherosclerosis are characterized by endothelial heterogeneity and dysfunction, where isolated or dysregulated cells lose the capacity to conduct hyperpolarizing signals or maintain nitric oxide bioavailability (Davignon and Ganz, 2004; Gimbrone and García-Cardeña, 2016). In both settings, the loss of electro-transcriptional identity may serve as an early predictor of vascular pathology.

Our work further raises the possibility of identifying novel markers of endothelial differentiation and dysfunction. High expression of FOXO3, MEF2C, Cx43, and KCa channels may define a mature, electrically competent endothelial state, while their loss could signify early maladaptive transitions. These markers could be exploited as diagnostic tools to detect endothelial vulnerability before overt vascular damage occurs. Moreover, restoring or stabilizing the transcriptional–electrical program pharmacologically (e.g., through SKCa/IKCa activators or FOXO3-inducing agents) may offer therapeutic potential in cardiovascular disease, cancer, or tissue repair.

Our study has several limitations that warrant consideration. We performed experiments in freshly isolated primary endothelial cell coupled maintained for only 48 h, a condition that minimizes contamination from other cell types and preserves responses that closely resemble those observed in intact vessels, as we previously reported (Burboa et al., 2024). Nonetheless, even short-term cultures cannot fully recapitulate the complex hemodynamic and structural environment of the intact vasculature. Moreover, while we improved the pharmacological design by employing selective blockers of hemichannels and gap junctions to differentiate isolated from coupled endothelial responses, we did not extend our validation to acutely dissociated endothelial cells. This remains an important next step to directly confirm the physiological relevance of the electrophysiological subpopulations identified.

Another limitation is that it remains unclear which specific connexin isoforms are primarily responsible for shaping the resting membrane potential profile. Carbenoxolone, although widely used, is a broad-spectrum connexin inhibitor, and therefore cannot discriminate between the contributions of different connexin subtypes. Identifying the connexin(s) and potential modifications that play the dominant role in establishing electrical heterogeneity will require additional genetic and molecular approaches.

Despite the valuable insights provided by *en face* arteriole preparations, these experiments were technically demanding, and tissue viability often limited reproducibility. Finally, although our data strongly support that endothelial coupling stabilizes membrane potential and enables KCa-driven hyperpolarization, the translational impact remains uncertain. In pathological conditions such as endothelial injury or chronic stress, arterioles may undergo junctional uncoupling, leading to loss of electrical integration and impaired vasomotor tone. Future studies in disease models will therefore be necessary to determine whether disruption of endothelial coupling represents a causal mechanism in vascular dysfunction.

In summary, our results uncover a multi-dimensional mechanism by which endothelial confluency controls electrical phenotype—not merely through physical junctions but via transcriptional regulation of ion channel expression. We propose that a specialized subset of hyperpolarized endothelial cells act as electrical hubs within the vascular network, coordinating local and conducted responses. Loss of this phenotype through inflammation, oxidative stress, isolation, or remodeling leads to electrical silencing, impaired signal conduction, and functional decline. Therapies aimed at preserving this transcriptional–electrical axis may represent a new frontier in vascular medicine, offering routes to prevent or reverse dysfunction in cardiovascular, oncological, and ischemic disease.

Notably, connexins should no longer be viewed as passive conduits for calcium or IP_3_ diffusion, but as active regulators of this synchronized electro-transcriptional orchestra. Their ability to maintain endothelial coherence at both the electrical and transcriptional levels appears essential for vascular homeostasis. For the connexin community, this poses an exciting challenge: to further dissect the molecular logic by which these proteins orchestrate multicellular identity and function across vascular beds.

## Methods

### Ethical approval

All animal procedures were conducted in accordance with the American Physiological Society’s Animal Care Guidelines, the National Institutes of Health (NIH) Guide for the Care and Use of Laboratory Animals, All protocols were reviewed and approved by the Institutional Animal Care and Use Committee (IACUC) at Rutgers University, Newark, NJ (PROTO202400075 and PROTO999900759).

### Mouse breeding

Wild-type (WT) mice were originally obtained from Jackson Laboratory and subsequently bred in our institutional animal facility. Experimental cohorts were evaluated between 4 and 6 months of age. All experiments were approved by the Rutgers New Jersey Medical School IACUC and carried out in full compliance with NIH animal care guidelines.

### Primary endothelial cell culture

Primary endothelial cells (ECs) were isolated from arterioles within the mesenteric vascular bed. Detailed procedures are described in (Burboa et al., 2024; Burboa et al., 2025; Younis et al., 2025).

### Measurement of intracellular Ca^2+^

Endothelial cells (ECs) were loaded with 5 µM Fluo-4AM and 0.02% (w/v) Pluronic F-127 in Tyrode’s solution buffered with 5 mM MOPS (pH 7.4), containing (in mM): 118 NaCl, 5.4 KCl, 2.5 CaCl_2_, 1.2 KH_2_PO_4_, 1.2 MgSO_4_, 23.8 NaHCO_3_, and 11.1 glucose. After 30 min of incubation, cells were washed and transferred to a temperature-controlled imaging chamber maintained at 37 °C. Fluo-4AM was dissolved in DMSO and diluted in MOPS-buffered Tyrode’s to the final working concentration. Fluorescence was recorded from 50 to 100 cells per coverslip (three coverslips per mouse), using four to five independent animals. For each experiment, a single average value was derived from the three coverslips. Changes in fluorescence were expressed as F/F_0_, where F is the recorded fluorescence and F_0_ is the baseline signal. Data were analyzed using nested one-way ANOVA.

### Pressure myography and endothelial membrane potential recordings

Mesenteric resistance arteries (second-to third-order, free of side branches) were isolated from anesthetized mice and mounted in a pressure myograph chamber (model 114P, Danish MyoTechnology A/S) using glass cannulas of 60–100 µm. Vessels were continuously superfused with physiological saline solution (PSS; in mmol/L: NaCl 118, KCl 4.7, CaCl_2_ 1.6, KH_2_PO_4_ 1.2, MgSO_4_ 1.2, NaHCO_3_ 25, glucose 5.5, HEPES 10; pH adjusted to 7.4) and imaged at 10 × magnification using a Zeiss AxioVert.A1 inverted microscope. Changes in outer and inner diameters were recorded and analyzed using MyoView software (v5.1). Active diameters were measured in standard PSS containing Ca^2+^, whereas passive diameters were obtained in Ca^2+^-free PSS supplemented with 2 mmol/L EGTA to abolish vascular tone. To mechanically disrupt the endothelium, a small air bubble was introduced into the vessel lumen. Under these conditions, endothelial membrane potential recordings were performed at a luminal pressure of 60 mmHg. In total, experiments were conducted in 15–20 mice, with 3–5 arterioles analyzed per animal.

Note that endothelial removal was tested using ACh and NO donors, as well as contractions induced by phenylephrine. Vessels that did not exhibit adequate vasomotor activity after endothelial removal were excluded from the analysis.

### Membrane potential recordings

Membrane potential was measured in intact smooth muscle cells (SMCs) from pressurized mesenteric resistance arteries (inner diameter 120–180 µm) and in intact endothelial cells (ECs) using sharp microelectrodes pulled from borosilicate glass capillaries and filled with 3 M KCl (tip resistance 30–60 MΩ). For EC recordings, *en face* arterial preparations were used to provide direct access to the endothelial layer. Microelectrodes were connected to a DUO 773 electrometer (World Precision Instruments, Sarasota, FL, United States), and vessels were pinned to a Sylgard-coated chamber and superfused with MOPS-buffered Tyrode’s solution (pH 7.4). To identify the impaled cell type, the electrode solution contained 10 µM Dextran-FITC (70 kDa). An Ag–AgCl reference electrode was used to complete the circuit. Successful impalement was indicated by a sharp negative voltage deflection upon penetration, a stable resting potential, and a positive shift upon withdrawal. Signals were sampled at 100 Hz using pClamp 10 (Molecular Devices, San Jose, CA, United States). Indomethacin was included to inhibit prostaglandin-mediated effects. In total, recordings were obtained from approximately 40–80 cells derived from 15 to 20 mice, with four arterioles analyzed per animal. Data were evaluated using nested one-way ANOVA.

Changes in membrane potential in both vessels and cells were evaluated according to the stability of the recording after impalement. After approximately 120–180 s, the average of the stable values obtained during this period was calculated and plotted in Figures 1–3. Recordings that remained negative after pipette withdrawal (i.e., after the impalement ended) were not included in the analysis.

### Proximity ligation assay (PLA)

The spatial distribution and interaction of S-nitrosylation with Cx43, as well as associations between Cx43/SKCa and Cx43/IKCa were assessed by PLA (MilliporeSigma). ECs were blocked and incubated with primary antibodies from different host species, followed by oligonucleotide-conjugated secondary antibodies per the manufacturer’s protocol. The antibodies used were: anti-Cx43 (cat. no. C8093, 1:200; MilliporeSigma), anti-IKCa (APC-064, 1:150, Alomone Labs) and anti-SKCa (cat. no. #APC-039,1:150 Alomone Labs). Dots were quantified using ImageJ, considering particles between 0.3 and 10 μm^2^ (Lillo et al., 2023; López-Cano et al., 2019). Data were obtained from 50 to 100 cells per coverslip (three coverslips per mouse, four to five mice), and analyzed by nested one-way ANOVA.

#### In situ hybridization

To detect the relative levels of transcription, we used the RNAscope® kit (Advanced Cell Diagnostics, ACD Catalog # 323136), following the protocol indicated by the manufacturer (ACD Document UM323100), with some modifications (ACD Technical Note MK50-012). Briefly, murine mesentery endothelial cell cultures (3 days *in vitro*) were prepared on glass coverslips, fed and subsequently fixed (4% PFA in PBS, 30 min at RT) and stored in PBS at 4 °C. Subsequently, the cultures were permeabilized and dehydrated by a gradient water/alcohol exposure, followed by drying (10 min) and subsequent rehydration in a gradient alcohol/water, all at RT. To ensure inactivation of ribonuclease activity, the cultures were exposed to RNAscope® hydrogen peroxide solution and RNAscope® Protease-III (included in the kit), to be finally stored in PBS at 4 °C. The next day, the cultures were incubated with specific probes for 2 h at 40 °C, using the ACD HybEZ™ II Hybridization System. Then, hybridization was amplified and detected by serial incubations and washes with amplifiers and fluorescent dyes included in the kit and specific for each probe. Finally, the samples were counterstained with DAPI solution (included in the kit) and mounted (ProLong® Diamond Antifade Media, Invitrogen). Images were acquired using a Nikon Ti2 HCA Inverted Fluorescence Microscope (Cellular Imaging and Histology Core, Rutgers-NJMS at Newark, NJ).

### Chemicals

All chemicals used were of analytical grade and obtained from MilliporeSigma (Billerica, MA, United States). Specifically, N^G-nitro-L-arginine methyl ester (L-NAME), MOPS, endothelial cell growth supplement from bovine pituitary, BSA, PE, ACh, GSK 1016790A, DEANO, charybdotoxin, and apamin were sourced from MilliporeSigma.

### Statistical analysis

Values are reported as mean ± S.E. Group comparisons were assessed using was assessed using nested one-way ANOVA as detailed by Eisner (Eisner, 2021). The significance level of P < 0.05 was considered statistically significant. Each figure legend specifies the respective sample size (n) and exact P value. Statistical analyses were performed using GraphPad Prism version 9.5.1 and Origin version 9.

## Data Availability

The original contributions presented in the study are included in the article, further inquiries can be directed to the corresponding author.
